# 10‐Gingerol alleviates hypoxia/reoxygenation‐induced cardiomyocyte injury through inhibition of the Wnt5a/Frizzled‐2 pathway

**DOI:** 10.1002/fsn3.2381

**Published:** 2021-06-05

**Authors:** Bin Zheng, Jiaying Qi, Panpan Liu, Muqing Zhang, Yuanyuan Zhang, Yucong Xue, Xue Han, Shan Xu, Li Chu

**Affiliations:** ^1^ School of Pharmacy Hebei University of Chinese Medicine Shijiazhuang China; ^2^ Affiliated Hospital Hebei University of Chinese Medicine Shijiazhuang China; ^3^ Hebei Key Laboratory of Chinese Medicine Research on Cardio‐cerebrovascular Disease Shijiazhuang China

**Keywords:** 10‐Gingerol, H9c2 cardiomyocyte, CoCl_2_, hypoxia/reoxygenation, intracellular Ca^2+^ overload, Wnt5a/Frizzled‐2 pathway

## Abstract

10‐Gingerol (10‐Gin), an active ingredient extracted from ginger, has been reported to have beneficial effects on the cardiovascular system. However, 10‐Gin has not been proved to offer protection against cardiomyocyte injury induced by hypoxia/reoxygenation (H/R). This study aimed to investigate the protective effects of 10‐Gin against H/R‐induced injury and its potential mechanisms in cardiomyocytes. A H/R injury model of H9c2 cardiomyocytes was established using 600 μmol/L CoCl_2_ to induce hypoxia in the cells for 24 hr and then reoxygenated for 3 hr. 10‐Gin was pretreated with H9c2 cardiomyocytes for 24 hr to assess its cardiomyocyte protection. Our results showed that 10‐Gin improved the viability of H9c2 cardiomyocytes in the H/R model and decreased the activities of creatine kinase, lactate dehydrogenase, and the generation of reactive oxygen species. By intracellular Ca^2+^ ([Ca^2+^]_i_) fluorescence, we found that 10‐Gin could significantly reduce the [Ca^2+^]_i_ concentration. 10‐Gin administration increased the activities of antioxidase and reduced malondialdehyde content and inflammatory cytokine levels. 10‐Gin also reduced the apoptosis levels. Importantly, 10‐Gin administration decreased the gene and protein expressions of Wnt5a and Frizzled‐2. In conclusion, 10‐Gin alleviates H/R‐induced cardiomyocyte injury, which is associated with the antioxidation, anti‐inflammation, antiapoptosis action, and reduction of [Ca^2+^]_i_ overload by suppressing the Wnt5a/Frizzled‐2 pathway.

## Graphical abstract

10‐Gingerol can attenuate the injury caused by hypoxia /reoxygenation reduce oxidative stress, Ca2+ overload, inflammation, and apoptosis, which is related to the inhibition of the Wnt5a/Frizzled‐2 pathway. Our research provides an experimental basis and new treatment strategies for MIRI. However, further research will be needed before these treatments can be used in clinics.

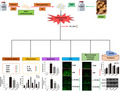

## 1 INTRODUCTION

Cardiovascular disease is a major cause of elevated human mortality, and its incidence is increasing (Aissaoui et al., 2020). Acute myocardial infarction (AMI) is a common cardiovascular disease that endangers human health (Najib et al., 2015). Previous studies have shown that AMI has high morbidity and mortality, and myocardial ischemia/reperfusion injury (MIRI) is an important cause of this condition (Herlitz et al., 2008; Najib et al., 2015). MIRI refers to an irreversible disease in which ischemic myocardial damage is aggravated after revascularization (Yellon & Hausenloy, 1997). MIRI is associated with a series of clinical problems, including thrombolysis, angioplasty, and coronary bypass surgery (Turer & Hill, 2010). With the application of reperfusion technology in AMI treatment, MIRI caused by reperfusion has become a hot topic in cardiovascular disease research. Therefore, there is an urgent need for new treatments for AMI to prevent and alleviate MIRI.

Currently, it is believed that the main pathogenesis of MIRI is the production of oxygen free radical (OFR) and the formation of intracellular Ca^2+^ ([Ca^2+^]_i_) overload (Turer & Hill, 2010). The generation of OFR is an important step in the progression toward MIRI, and [Ca^2+^]_i_ overload is the terminal link of irreversible injury (Tani, 1990; Turer & Hill, 2010). Excess OFR can cause severe oxidative stress damage, leading to the abnormal function of the endoplasmic reticulum and [Ca^2+^]_i_ overload (Jennings, 2013). Moreover, inflammatory and oxidative stress are closely linked and are characteristics of cardiac diseases (Cachofeiro et al., 2008). During MIRI, oxidative stress causes endoplasmic reticulum damage and reactivation of mitochondria (Jennings, 2013). The mitochondrial membrane potential (MMP) is restored, and Ca^2+^ is driven into the mitochondria through the single transporter, and the mitochondrial permeability transition pore (mPTP) is induced to open (Weiss et al., 2003). As a result, the homeostasis of [Ca^2+^]_i_ is destroyed, resulting in increased Ca^2+^ influx, unbalanced free Ca^2+^ reuptake, and Ca^2+^ accumulation in the cytoplasm (Weiss et al., 2003). The significantly increased free Ca^2+^ concentration in the cytoplasm will eventually lead to cell necrosis, apoptosis, enhanced autophagy, and cell degeneration (Aldakkak et al., 2011; Eefting et al., 2004).

The Wnt/Frizzled signaling pathway is considered the regulation target of various cardiovascular diseases (Blankesteijn et al., 1997). It has been proven that after myocardial infarction, the Wnt/Frizzled pathway is activated in the process of myocardial reconstruction and heart failure (Sheldahl, Park, Miller & Moon., 2000). Wnt5a is believed to be closely associated with [Ca^2+^]_i_ metabolism, and its activation can trigger [Ca^2+^]_i_ release through the Wnt/Ca^2+^ pathway. Importantly, the above effects of Wnt5a are realized through the activation of the Frizzled‐2 receptor (Slusarski and Corces et al., 1997). Furthermore, the overexpression of Frizzled‐2 can activate the Wnt5a/Frizzled‐2 pathway, thereby inducing the accumulation of Ca^2+^, and this effect can be reversed by inhibiting Frizzled‐2 (Zhou et al., 2012).

**FIGURE 1:**
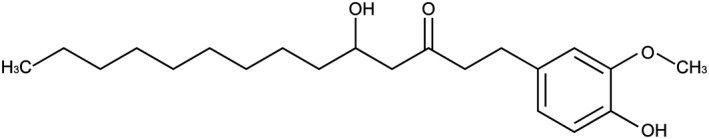
Chemical structure formula of 10‐Gin

Ginger (*Zingiber officinale Roscoe, Zingiberaceae*), a perennial herb of the zingiberaceae family, is native to South Asia, and it has long been used as a daily spice flavoring around the world (Ghareib et al., 2015). People in ancient Greece, China, and India have used ginger to treat various diseases (Haniadka et al., 2012). Ginger contains a variety of phenolic components that have been shown to possess biological activities, including antioxidation, antitumor, anti‐inflammatory, and antiglycemic activities (Ali et al., 2008; Bode et al., 2001; Katiyar et al., 1996; Minghetti et al., 2007; Shukla & Singh, 2007). In our previous studies, we have demonstrated that 6‐Gin, a component from ginger, protects against myocardial fibrosis (Han et al., 2020) and can significantly inhibit L‐type Ca^2+^ current and alleviate myocardial ischemia in vivo and in vitro (Han et al., 2019). However, 10‐Gin (C_21_H_34_O_4_; Figure 1), the main ingredient in gingerol and similar to 6‐Gin, has not been shown to have a protective effect on MIRI, and its underlying mechanism has not been studied.

We hypothesized that 10‐Gin offers protection to H9c2 cardiomyocyte injury induced by hypoxia/reoxygenation (H/R). In our study, the protection of 10‐Gin was determined by detecting the viability of H9c2 cells and the levels of oxidative stress, [Ca^2+^]_i_ concentration, inflammation, and apoptosis in the H/R model. Furthermore, the underlying mechanisms were explored by detecting the protein and gene expression levels of the Wnt5a/Frizzled‐2 pathway. Our research provides new strategies and methods for mitigating MIRI in the treatment of AMI.

## 2 MATERIALS AND METHODS

### 2.1 Reagents

10‐Gin (Biopurify, China, Catalog: BP0013, HPLC ≥98%) was dissolved in dimethyl sulfoxide to prepare a 100 mmol/L stock solution. All kits used in this experiment were purchased from the Jiancheng Bioengineering Institute of Nanjing (Nanjing, China). High‐glucose Dulbecco's modified Eagle's medium (DMEM, Catalog: 12,430,047), fetal bovine serum (FBS, Catalog: 10,093,188), trypsin (Catalog: 25,200,072), and penicillin/streptomycin were purchased from Gibco (Thermo Fisher Scientific, Inc. Missouri, USA). Unless otherwise stated, the other chemical reagents of analytical grade were obtained from Sigma‐Aldrich (Missouri, USA).

### 2.2 Cell culture

The rat H9c2 cardiomyoblasts (Bluefbio, Shanghai, China, Catalog: BFN60804388) were cultured in high‐glucose DMEM containing 10% FBS and 100 U/mL penicillin/streptomycin in 5% CO_2_ and 95% air at 37 ℃. The medium was changed every 2 days. The cells were digested with 0.25 g/L trypsin and counted when the degree of cell fusion was about 80%. The cells were inoculated into a 25‐cm^2^ culture flask for passage every 3 days. H9c2 cardiomyocytes at the logarithmic growth stage were taken for subsequent experiments.

### 2.3 Establishment of the H/R model

CoCl_2_ (Catalog: 449,776) is a common chemical anoxic reagent that can simulate ischemic/hypoxia conditions in various cells (Jung et al., 2007). H9c2 cardiomyocytes were prepared into 5 × 10^4^/ml cell suspension and inoculated in a 96‐well plate at 100 μL/well. After the cells were incubated in an incubator containing 5% CO_2_ at 37 ℃ for 24 hr, the cells were treated with hypoxia when the degree of cell fusion reached 80%–100%. CoCl_2_ solutions with different concentrations (200, 400, 600, 800, and 1,000 μmol/L) were prepared with high‐glucose DMEM and incubated for 24 hr, respectively, and the optimal concentration of hypoxia was determined according to the results of cell counting kit‐8 (CCK‐8, Catalog: G021‐1–1). After hypoxia had ended, the anoxic solution containing CoCl_2_ was discarded, and the cells were washed three times with phosphate‐buffered saline (PBS, Catalog: 003,002). Then, the high‐glucose DMEM containing 10% FBS was used for reoxygenation for 1, 2, 3, 4, 5, and 6 hr, respectively. According to the results of the CCK‐8 kits, the optimal reoxygenation time was determined. Based on the above results, the H/R model was established.

### 2.4 Experimental treatment

H9c2 cardiomyocytes were randomly divided into the four groups: the control group (CONT, normal cultured cells); the H/R group (H/R, after 24‐hr incubation with 600 μmol/L of CoCl_2_ solution, the anoxic solution was discarded, and the culture was continued in normal medium for 3 hr); the low‐dose 10‐Gin pretreatment group (L‐10‐Gin, cells were pretreated with 10‐Gin at 10 μmol/L for 24 hr, other steps are consistent with the H/R group); and the high‐dose 10‐Gin pretreatment group (H‐10‐Gin, cells were pretreated with 10‐Gin at 30 μmol/L for 24 hr, other steps are consistent with the H/R group).

### 2.5 Detection of H9c2 cardiomyocyte activity by CCK‐8 assay

The CCK‐8 assay was used to detect the effects of 10‐Gin (10, 20, 30, 40, 50, and 60 μmol/L) on the viability of H9c2 cardiomyocytes cultured in 96‐well plates. Briefly, according to the manufacturer's instructions, when the cells had grown to 80% confluence, different concentrations of 10‐Gin were added into the plate treated for 24 hr in 5% CO_2_ and 95% air at 37 ℃. Then, 100 µl/well culture medium and 10 µl/well CCK‐8 solution were added to each well, and the cells were incubated for a further 2 hr at 37 ℃. Absorbance was measured at 450 nm with an enzyme labeling instrument (Thermo Scientific Varioskan LUX, Thermo Fisher Scientific, Inc.). The hypoxia concentration of CoCl_2_ was determined as 600 μmol/L, and the concentrations of the L‐10‐Gin group and H‐10‐Gin group were determined to be 10 μmol/L and 30 μmol/L, respectively. Then, according to the experimental treatment, the viability of H9c2 cardiomyocytes in each group was detected after the experimental procedure.

### 2.6 Assessment of lactated dehydrogenase (LDH) and creatine kinase (CK)

After the experimental procedure, the cell culture supernatant of the four groups was collected and centrifuged at 645 × g for 10 min. After centrifugation, 100 μL/well of supernatant was added in 96‐well plates, and the activities of LDH (Catalog: A020‐2–2) and CK (Catalog: A032‐1–1) in each group were detected according to the kit instructions.

### 2.7 Detection of oxidative stress levels

After the experimental treatments, the cells were digested with an appropriate amount of trypsin and centrifuged at 645 × g for 10 min. The supernatant was discarded, and 500 μL PBS solution was added to the cell precipitation to resuspend and mix. Then, the cells were ruptured under the condition of ultrasound for 2 s and repeated ten times to obtain a crushed cell homogenate. The levels of superoxide dismutase (SOD, Catalog: A001‐3–2), catalase (CAT, Catalog: A007‐1–1), glutathione (GSH, Catalog: A006‐2–1), and malondialdehyde (MDA, Catalog: A003‐1–1) in the cell homogenate were determined according to the instructions of the kit.

### 2.8 Measurement of reactive oxygen species (ROS) generation

2,7‐Dichlorodihydrofluorescein diacetate (DCFH‐DA, Cayman Chemical, Michigan, USA, Catalog: 85,155) is a common dichlorofluorescein used as an indicator of peroxynitrite formation. Briefly, after the experimental procedure, the supernatant of cultured cells in a 24‐well plate was discarded, and 500 μL/well of DCFH‐DA dye was added into each group. The cells were incubated in the dark at 37 ℃ for 20 min. Next, the dye was removed, and the cells were twice rinsed with PBS, and a fluorescence microscope was used for fluorescence image collection. Image‐Pro Plus 6.0 software (Media Cybernetics, Inc.) was used for quantification.

### 2.9 Analysis of [Ca^2+^]_i_ concentration by Fluo‐3 a.m. staining

The cells were inoculated in 24‐well plates, and the Fluo‐3 a.m. (Catalog: F1242) concentration was diluted to 10 μmol/L according to the instructions of the Ca^2+^ GPCR assay calcium ion indicating probe kit. Then, 500 μL/well of dye was added to each well and incubated at 37 ℃ in the dark for 20 min. Next, the dye was removed, and the cells were rinsed with PBS twice, and a fluorescence microscope was used for fluorescence image collection. Image‐Pro Plus 6.0 software was used for quantification.

### 2.10 Assay of MMP by Rhodamine 123 (Rh123) staining

Cell mitochondria can rely on their membrane electronegative to assimilate the cationic fluorescent dyeing Rh123 (Shanghai Yuanye Biotechnology, Shanghai, China, Catalog: S19123). Damage to a cell's MMP can result in reduced uptake capacity of Rh123. Therefore, the mitochondrial function can be indirectly inferred from the intake of Rh123. Briefly, after the experimental procedure, the supernatant of cultured cells in a 24‐well plate was discarded, and 500 μL/well of Rh123 (10 μg/mL) dyeing was added into each group. The cells were incubated in the dark at 37 ℃ for 15 min. Next, the dye was removed, and the cells were rinsed twice with PBS, and a fluorescence microscope was used for fluorescence image collection. Image‐Pro Plus 6.0 software was used for quantification.

### 2.11 Detection of intracellular inflammatory cytokines

After the experimental treatments, the cells were digested with an appropriate amount of trypsin and centrifuged at 645 × g for 10 min. The supernatant was discarded, and 500 μL PBS solution was added to the cell precipitation to resuspend and mix. Then, the cells were ruptured under the condition of ultrasound for 2 s and repeated ten times to obtain the crushed cell homogenate. The crushed cell homogenate was collected as samples to be tested, and the levels of interleukin‐6 (IL‐6, Catalog: H007), interleukin‐1β (IL‐1β, Catalog: H002), and tumor necrosis factor‐α (TNF‐α, Catalog: H052) were determined according to the kit instructions.

### 2.12 Assessment of apoptosis by flow cytometry and Hoechst‐33258 staining

An Annexin V‐FITC/PI apoptosis detection kit (Catalog: G003‐1–3) was used to detect cell apoptosis. After the experimental procedure, the cells of the different treatment groups were collected and washed three times with PBS. Then, the cells were resuspended with a prepared binding buffer (100 μL) and adjusted to a density of 1 × 10^6^ /ml. 100 μL of cell suspension was inoculated into a 5‐mL flow tube and stained with 5 μL Annexin V‐FITC and 5 μL PI that incubate away from light at room temperature for 15 min. Finally, the apoptosis rate was analyzed by flow cytometry (Beckman FC500, Beckman Coulter, Inc.).

Hoechst‐33258 (Beijing Solarbio Science & Technology, Beijing, China, Catalog: IH0060) is a specific DNA dye that can detect cell apoptosis through fluorescence intensity. After the experimental procedure, the cell culture medium of 24‐well plates in each group was discarded, and H9c2 cardiomyocytes were washed three times with PBS. Then, 500 μL/well of Hoechst‐33258 staining solution (10 μg/mL) was added to each group of cells and incubated at 37 ℃ for 20 min. Next, the dye was removed, and the cells were rinsed twice with PBS, and a fluorescence microscope was used for fluorescence image collection. Image‐Pro Plus 6.0 software was used for quantification.

### 2.13 Measurement of Caspase‐3 activity

After the experimental procedure, the supernatant of each treatment group was discarded, and the lysate provided by the kit was added, and the cells of each group were incubated on ice for 15 min. Then, the cells were centrifuged at 12,000 × g for 15 min. The supernatant was collected and stored at −80 ℃. In strict accordance with the kit's instructions, the protein samples were mixed with the substrate in a 37 ℃ water bath for 1 hr, and the absorbance of the fluorescent substrate was determined at 405‐nm wavelength. The activity of caspase‐3 (Catalog: G015‐1–3) was calculated according to the kit instructions.

### 2.14 Real‐time polymerase chain reaction (RT‐PCR)

SYBR Green Ⅰ RT‐PCR was used to detect the changes in the mRNA transcription level of the target gene in the samples. Total RNA was extracted from cell samples using TRIzol (Ambion, Thermo Fisher Scientific, Inc., Catalog: 15,596,018), and 1 μL RNA was taken to measure the quality and concentration of RNA and to detect RNA integrity. Reverse transcription was carried out in a volume of 20 µl containing 1 µg total RNA. The expression of Wnt5a, Frizzled‐2, and β‐actin in H9c2 cells was quantitatively detected by quantitative fluorescence PCR, and the relative quantitative analysis of the data was carried out by the 2^‐∆∆CT^ method. The PCR conditions were as follows: 40 cycles at 95 ℃ for 1 min, 95 ℃ for 15 s, and 55 ℃ for 1 min. β‐Actin was used as an internal reference. The primer sequence is shown in Table 1.

**TABLE 1:** Detection of target gene primers by real‐time PCR

Primer	Sequences (5’→3’)
Wnt5a	Forward: GGACTTACCTCGGGACTGG
	Reverse: CGACCTGCTTCATTGTTGTG
Frizzled−2	Forward: GCAGGGCACTAAGAA AGAA
	Reverse: ACCAGGTGAGGGACAGAA
β‐actin	Forward: CGTTGACATCCGTAAAGAC
	Reverse: TAGGAGCCAGGGCAGTA

### 2.15 Western blot analysis

The protein expression levels of Wnt5a and Frizzled‐2 were measured by Western blotting. After the experimental procedure, cells in each group were rinsed three times with precooled PBS, and RIPA lysate (Beijing Solarbio Science & Technology, Beijing, China, Catalog: R0020) was added and incubated overnight at 4 ℃, then centrifuged at 12,000 × g for 10 min. The supernatant was taken, and the Wnt5a, Frizzled‐2, and β‐actin protein contents in the sample were detected by Coomassie Brilliant Blue Reagent Kit (Shanghai Yuanye Biotechnology, Shanghai, China, Catalog: R21271). Protein buffer was added for protein denaturation and electrophoresis. The separated protein bands were fixed on polyvinylidene difluoride membrane (Millipore Sigma, Inc., Catalog: IPVH00010), and the transfer times were as follows: Frizzled‐2, 45 min; Wnt5a, 37 min; and β‐actin, 37 min. The membrane was then sealed with skimmed milk, then reacted overnight at 4 ℃ with rabbit anti‐Wnt5a (Affinity Biosciences, Jiangsu, China; diluted at 1:1,000, Catalog: DF6856), rabbit anti‐Frizzled‐2 (Affinity Biosciences, Jiangsu, China; diluted at 1:500, Catalog: AF5282), and rabbit anti‐β‐actin (Affinity Biosciences, Jiangsu, China; diluted at 1:1,000, Catalog: AF7018), respectively. After washing three times with 1 × PBST, the horseradish peroxidase‐labeled secondary diluted antibodies (1:3,000) were incubated at room temperature for 90 min to develop color. The Tanon GIS software measured the gray value of the strip (Tanon, Inc., Shanghai, China) after the film was scanned.

### 2.16 Statistical analyses

The measurement values are shown as the mean ±standard error of the mean (*SEM*), and the differences in data among groups were analyzed via one‐way analysis of variance (ANOVA) followed by Tukey's test using the Origin 7.5 software (OriginLab, Inc.). *P*‐values < 0.05 were considered statistically significant.

## 3 RESULTS

### 3.1 Effects of 10‐Gin on cell viability

**FIGURE 2:**
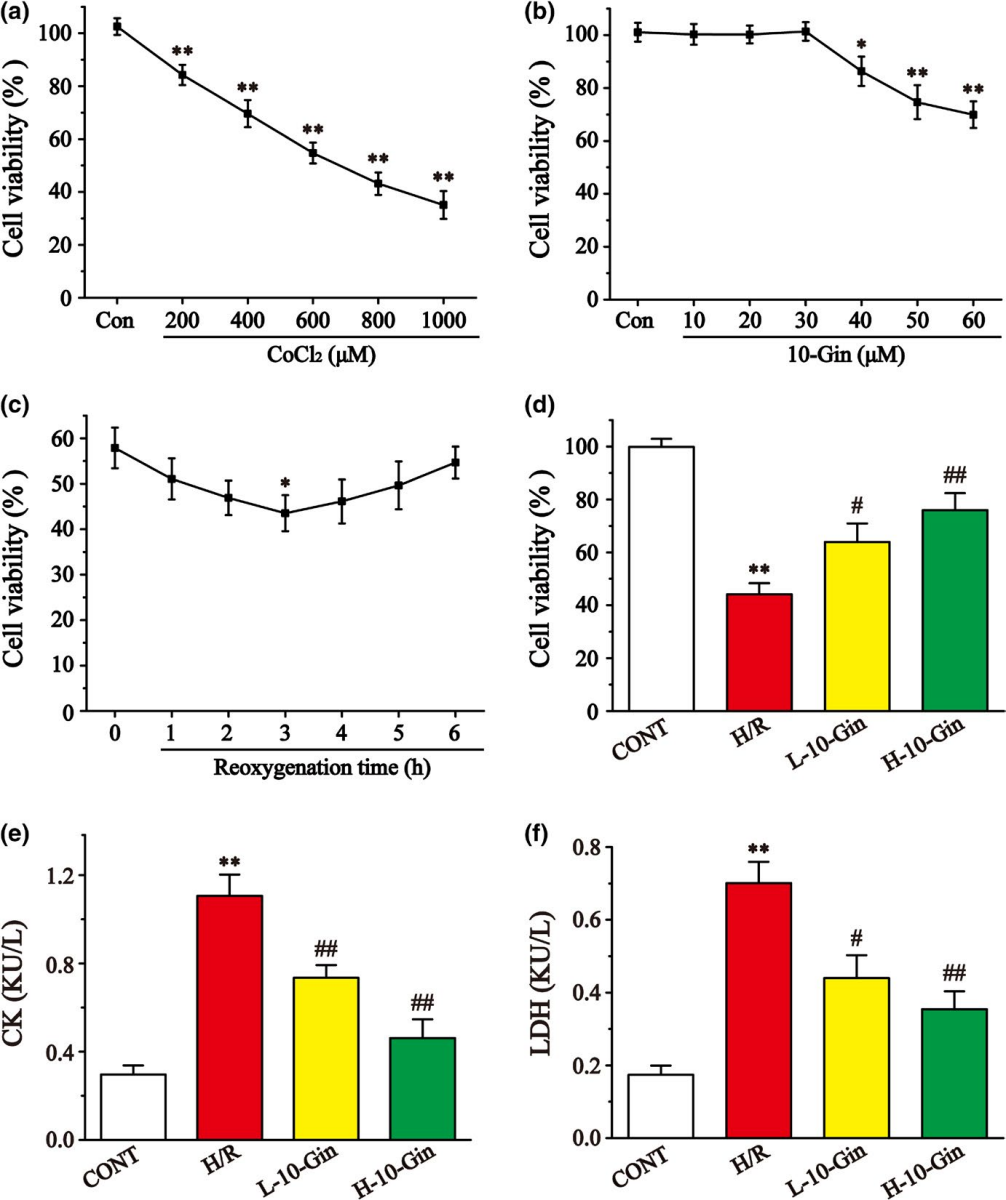
Effects of CoCl_2_ and 10‐Gin on the vitality of H9c2 cells by CCK‐8 assay, and effects of 10‐Gin on CK (e) and LDH (f). H9c2 cells were incubated with different concentrations of CoCl_2_ (a) and 10‐Gin (b) for 24 hr, followed by reoxygenation (c). The vitality of H9c2 cells in each group is shown by the CCK‐8 assay (d). Data are represented as mean ± *SEM*. * *p* <.05, ** *p* <.01 versus. CONT; ^#^ *p* <.05, ^##^ *p* <.01 versus. H/R group, *n* = 6

The results of H9c2 cardiomyocyte cytotoxicity show that 600 μmol/L CoCl_2_ and 40 μmol/L‐10‐Gin could significantly inhibit the viability of the H9c2 cells (Figure 2a and 2b) (*p* <.05 or *p* <.01). Therefore, 600 μmol/L CoCl_2_ (Figure 2a) was determined as the hypoxia concentration, and 10 μmol/L and 30 μmol/L‐10‐Gin (Figure 2b) were determined as the low‐ and high‐dose group concentrations, respectively. Figure 2c shows that the cell viabilities were in a U‐shape, indicating that the maximum damage occurred at the third hour after reoxygenation. Figure 2d shows that the cell viability in the H/R group was obviously reduced compared with the CONT group (*p* <.01). But 10‐Gin treatment could reverse the situation compared with the H/R group, showing that 10‐Gin could protect against the cardiomyocyte damage induced by H/R (*p* <.05 or *p* <.01).

### 3.2 Effects of 10‐Gin on CK and LDH

Figure 2e and 2f shows that the levels of CK and LDH were detected to assess H9c2 cardiomyocyte injury induced by H/R. Compared with the CONT group, the activities of CK and LDH in the H/R group were obviously increased (*p* <.01). But 10‐Gin treatment significantly decreased the activities of CK and LDH compared with the H/R group, suggesting that pretreatment with 10‐Gin can reduce the cardiomyocyte damage induced by H/R (*p* <.05 or *p* <.01).

### 3.3 Effects of 10‐Gin on oxidative stress

**FIGURE 3:**
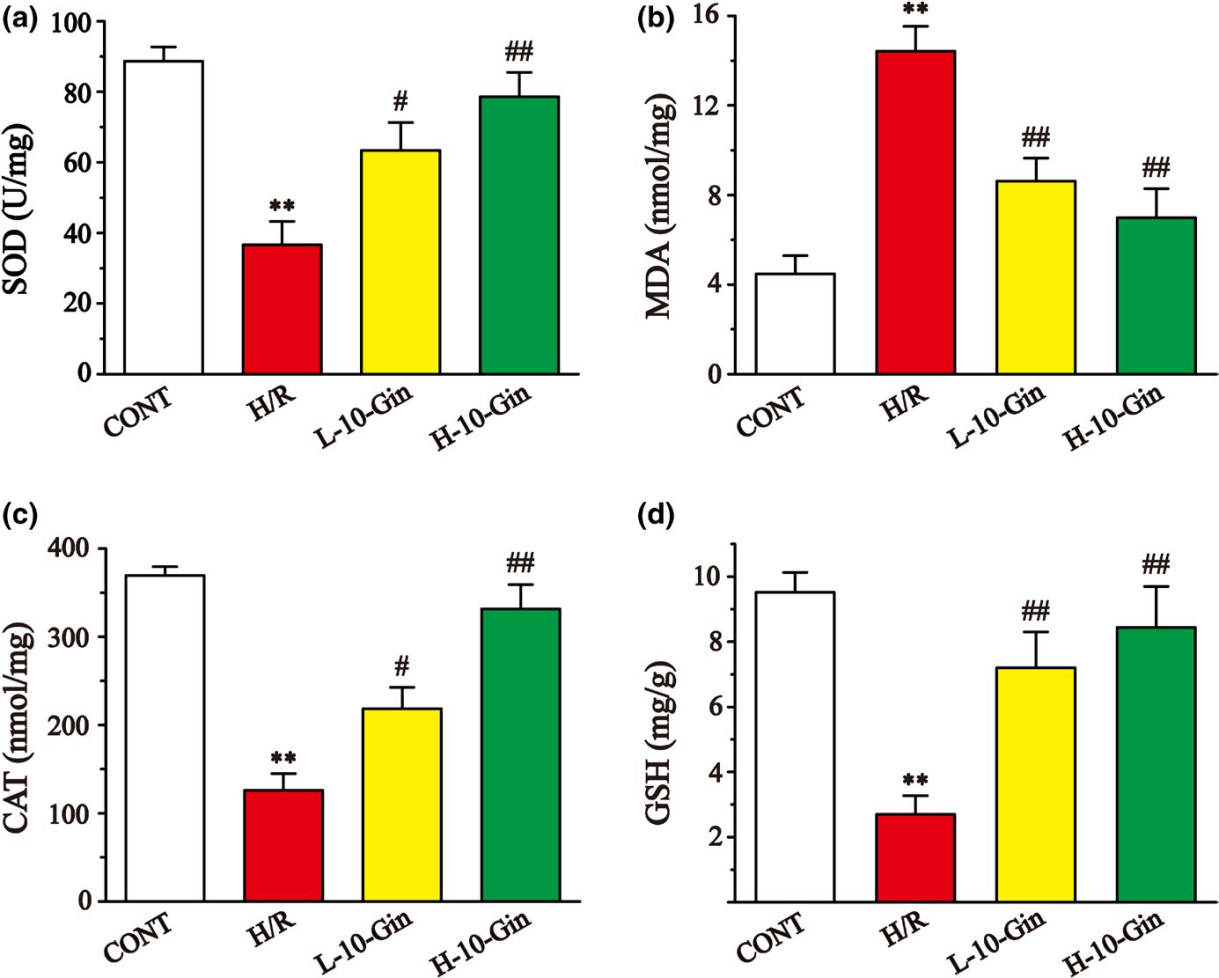
Effects of 10‐Gin on the levels of SOD (a), MDA (b), CAT (c), and GSH (d). Data are represented as mean ± *SEM*. ** *p* <.01 versus. CONT; ^#^ *p* <.05, ^##^ *p* <.01 versus. H/R group, *n* = 6

The levels of SOD, CAT, GSH, and MDA are shown in Figure 3. The levels of SOD, CAT, and GSH in the H/R group were lower than the CONT group (*p* <.01), while the content of MDA was higher (*p* <.01). However, the levels of SOD, CAT and GSH in the 10‐Gin treatment group were obviously increased compared with the H/R group (*p* <.05 or *p* <.01), while the levels of MDA were observably decreased (*p* <.01), showing that 10‐Gin could protect against oxidative stress injury induced by H/R.

### 3.4 Effects of 10‐Gin on ROS generation

**FIGURE 4:**
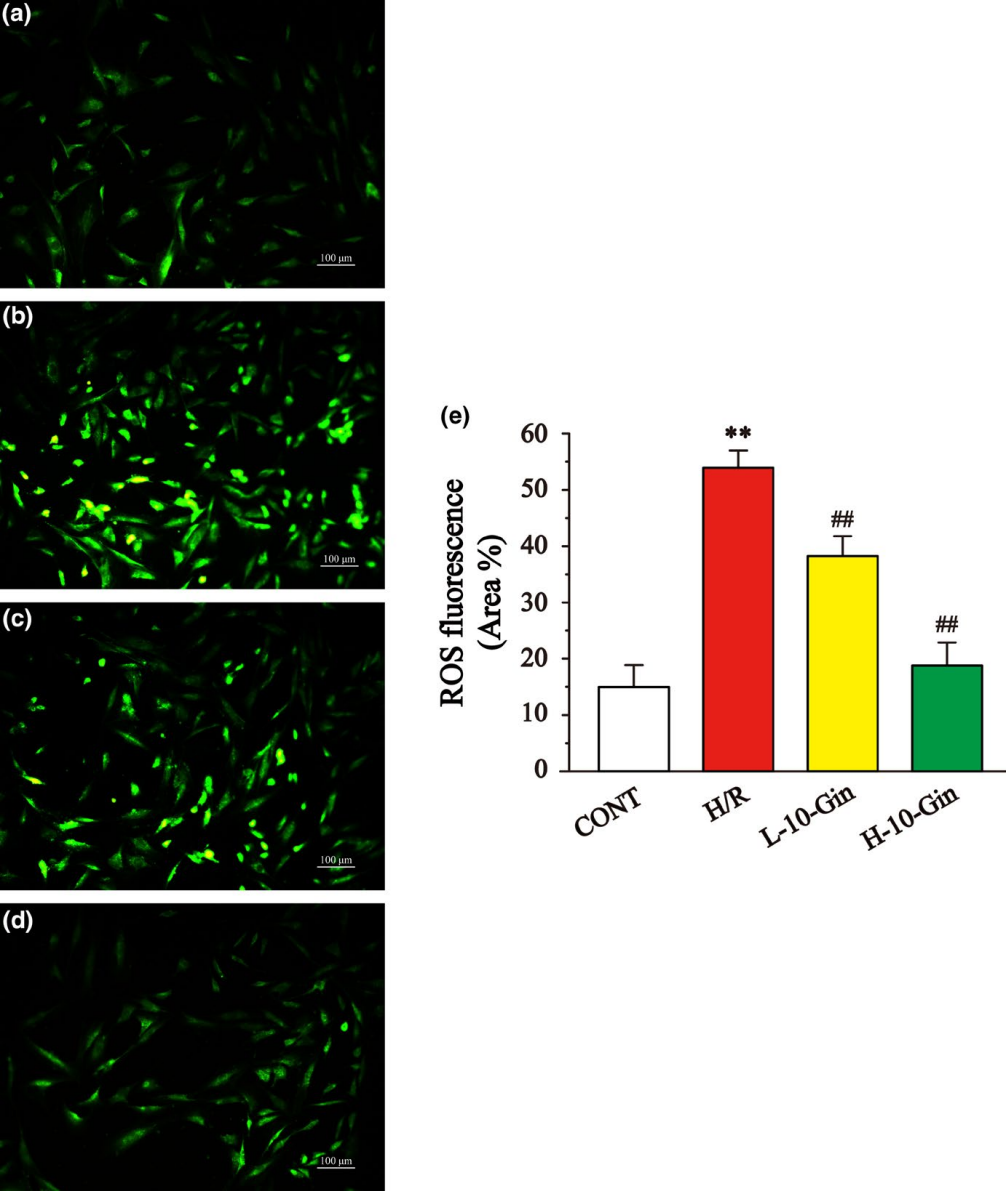
Effects of 10‐Gin on levels of ROS. ROS fluorescence images of different groups (a) CONT group; (b) H/R group; (c) L‐10‐Gin treatment group; and (d) H‐10‐Gin treatment group) and the ratio graph E about ROS fluorescence intensity are presented. Scale bar =100 µm (200 ×). Data are represented as mean ± *SEM*. ** *p* <.01 versus. CONT; ^##^ *p* <.01 versus. H/R group, *n* = 6

Figure 4 shows the protective effect of 10‐Gin on H/R‐induced H9c2 cardiomyocyte injury through measuring ROS levels. Compared with the CONT group, ROS levels in the H/R group were significantly increased (*p* <.01). However, the ROS levels in the 10‐Gin treatment group were significantly lower than the H/R group (*p* <.01). These results suggest that 10‐Gin can play a protective role by reducing the generation of ROS.

### 3.5 Effects of 10‐Gin on [Ca^2+^]_i_ concentration

**FIGURE 5:**
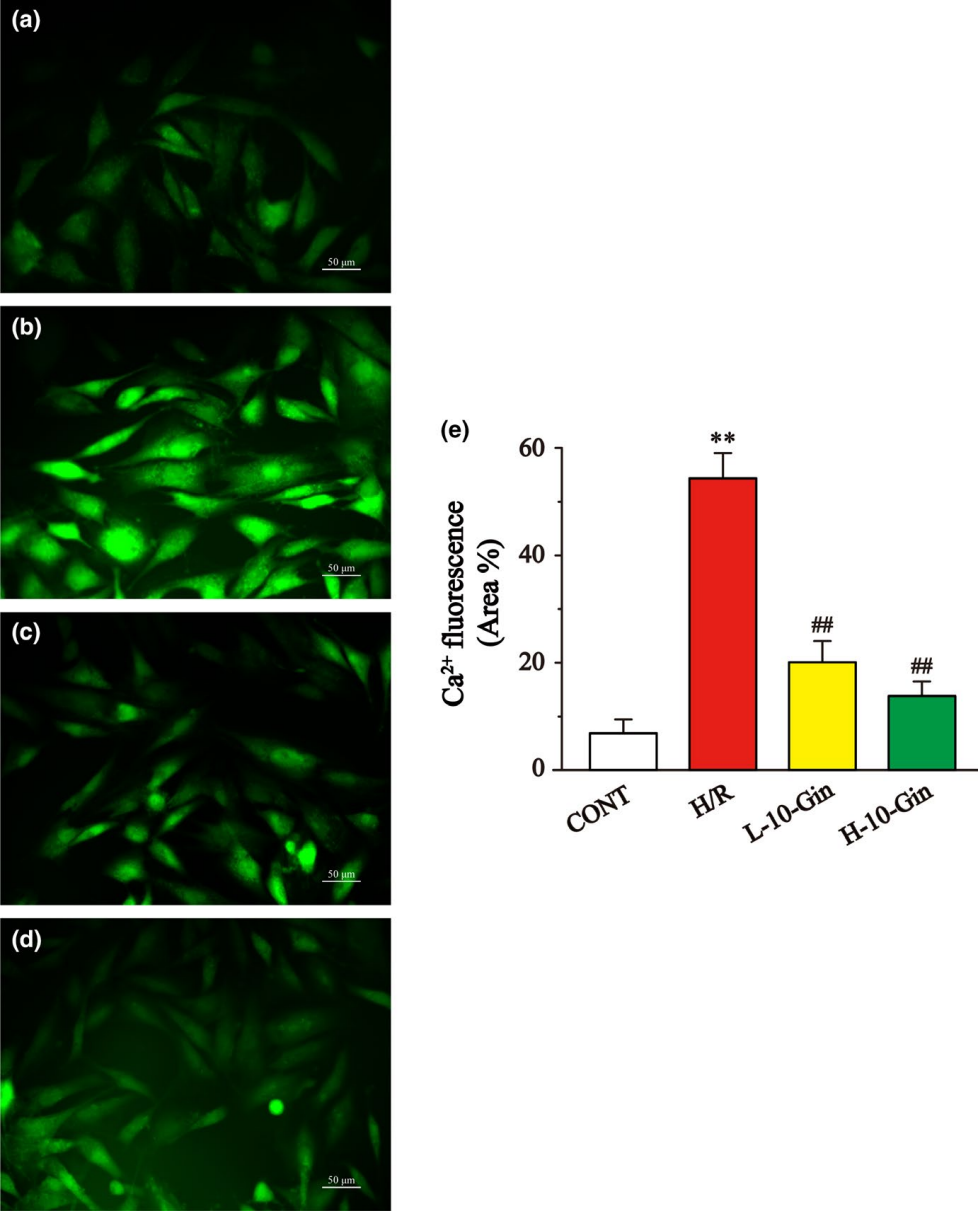
Effects of 10‐Gin on [Ca^2+^]_i_ concentration of H/R cardiomyocytes by flow‐3 staining. [Ca^2+^]_i_ fluorescence images of different groups (a) CONT group; (b) H/R group; (c) L‐10‐Gin treatment group; and (d) H‐10‐Gin treatment group) and the ratio graph (e) about [Ca^2+^]_i_ fluorescence intensity are presented. Scale bar =50 µm (400 ×). Data are represented as mean ± *SEM*. ** *p* <.01 versus. CONT; ^##^ *p* <.01 versus. H/R group, *n* = 6

The results of Figure 5 show that the [Ca^2+^]_i_ concentration in the H/R group was significantly higher than that in the CONT group (*p* <.01). However, the 10‐Gin treatment significantly decreased the concentration of [Ca^2+^]_i_ (*p* <.01). These results suggest that 10‐Gin has a protective effect on cell injury induced by H/R, and it can be achieved by inhibiting the [Ca^2+^]_i_ concentration.

### 3.6 Effects of 10‐Gin on MMP

**FIGURE 6:**
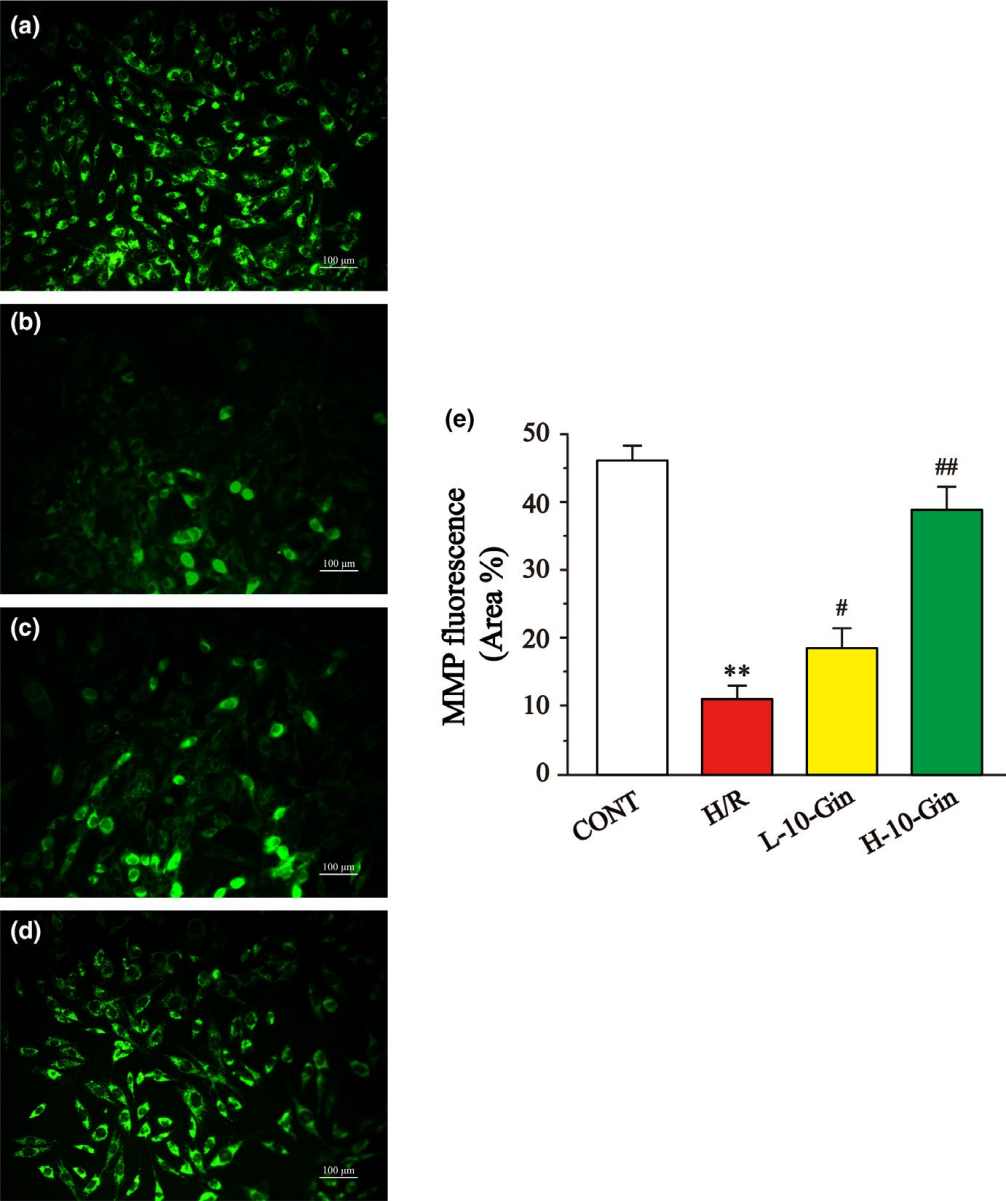
Effects of 10‐Gin on the MMP. Fluorescence images of different groups (a) CONT group; (b) H/R group; (c) L‐10‐Gin treatment group; and (d) H‐10‐Gin treatment group) and the ratio graph (e) about fluorescence intensity are presented. Scale bar =100 µm (200 ×). Data are represented as mean ± *SEM*. ** *p* <.01 versus. CONT; ^#^ *p* <.05, ^##^ *p* <.01 versus. H/R group, *n* = 6

Mitochondria are sensitive to noxious irritation, and their functional status can be reflected by detecting its uptake of fluorescent dye Rh123. As shown in Figure 6, compared with the CONT group, the uptake ability of Rh123 in H9c2 cardiomyocytes of the H/R group was significantly reduced (*p* <.01), suggesting that intracellular mitochondrial was damaged. In contrast, the uptake ability of Rh123 in H9c2 cardiomyocytes of the 10‐Gin treatment group was obviously increased relative to the H/R group (*p* <.05 or *p* <.01). These results indicate that 10‐Gin could reduce the mitochondrial damage caused by H/R and improve mitochondrial function.

### 3.7 Effects of 10‐Gin on inflammatory cytokines

**FIGURE 7:**
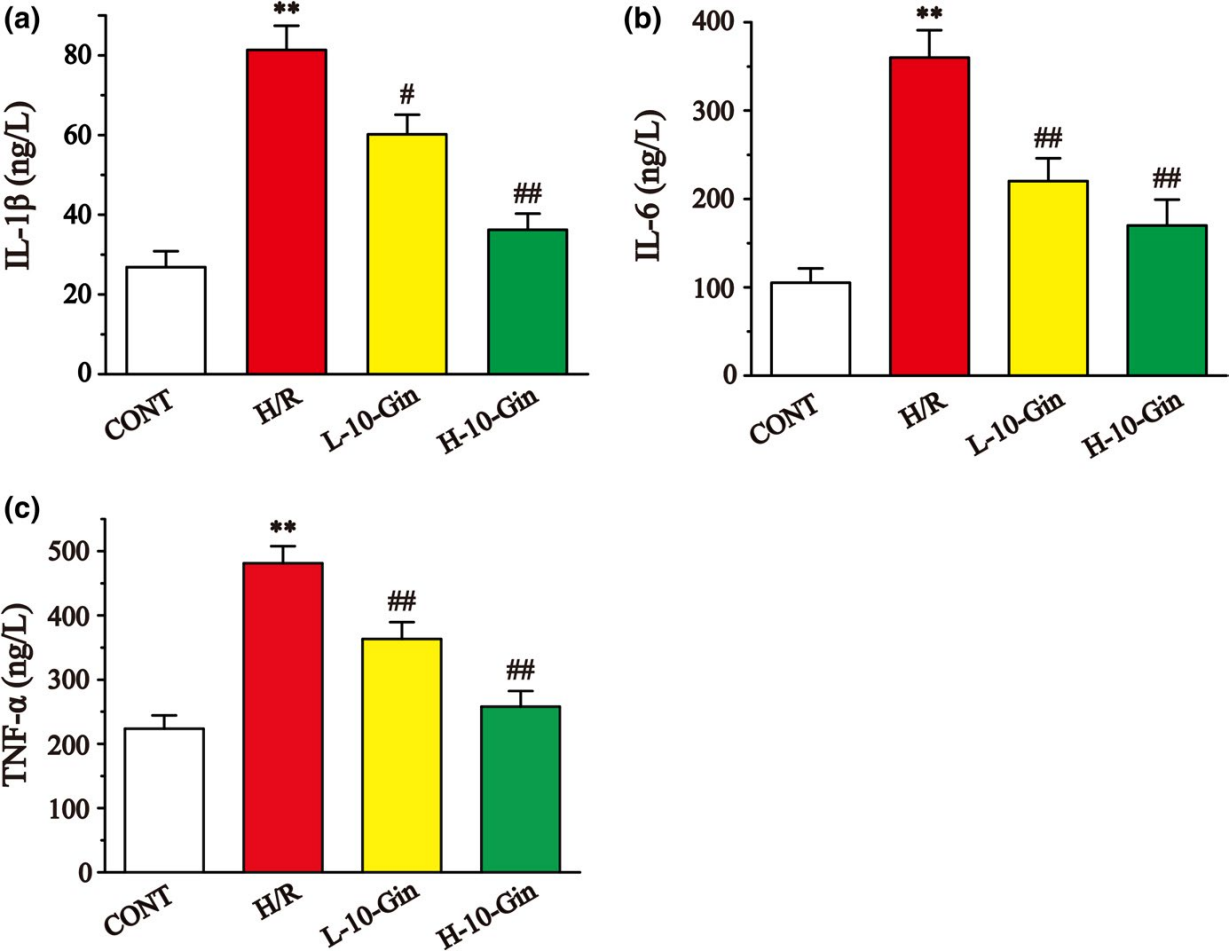
Effects of 10‐Gin on the levels of IL‐1β (a), IL‐6 (b), and TNF‐α (c). Data are represented as mean ± *SEM*. ** *p* <.01 versus. CONT; ^#^ *p* <.05, ^##^ *p* <.01 versus. H/R group, *n* = 6

Figure 7 shows the levels of IL‐1β, IL‐6, and TNF‐α. Compared with the CONT group, the levels of IL‐1β, IL‐6, and TNF‐α in the H/R group were markedly upregulated (*p* <.01). However, 10‐Gin treatment significantly reduced the levels of IL‐1β, IL‐6, and TNF‐α compared with the H/R group (*p* <.05 or *p* <.01). These results indicate that 10‐Gin could reduce the inflammatory injury caused by H/R.

### 3.8 Effects of 10‐Gin on apoptosis

**FIGURE 8:**
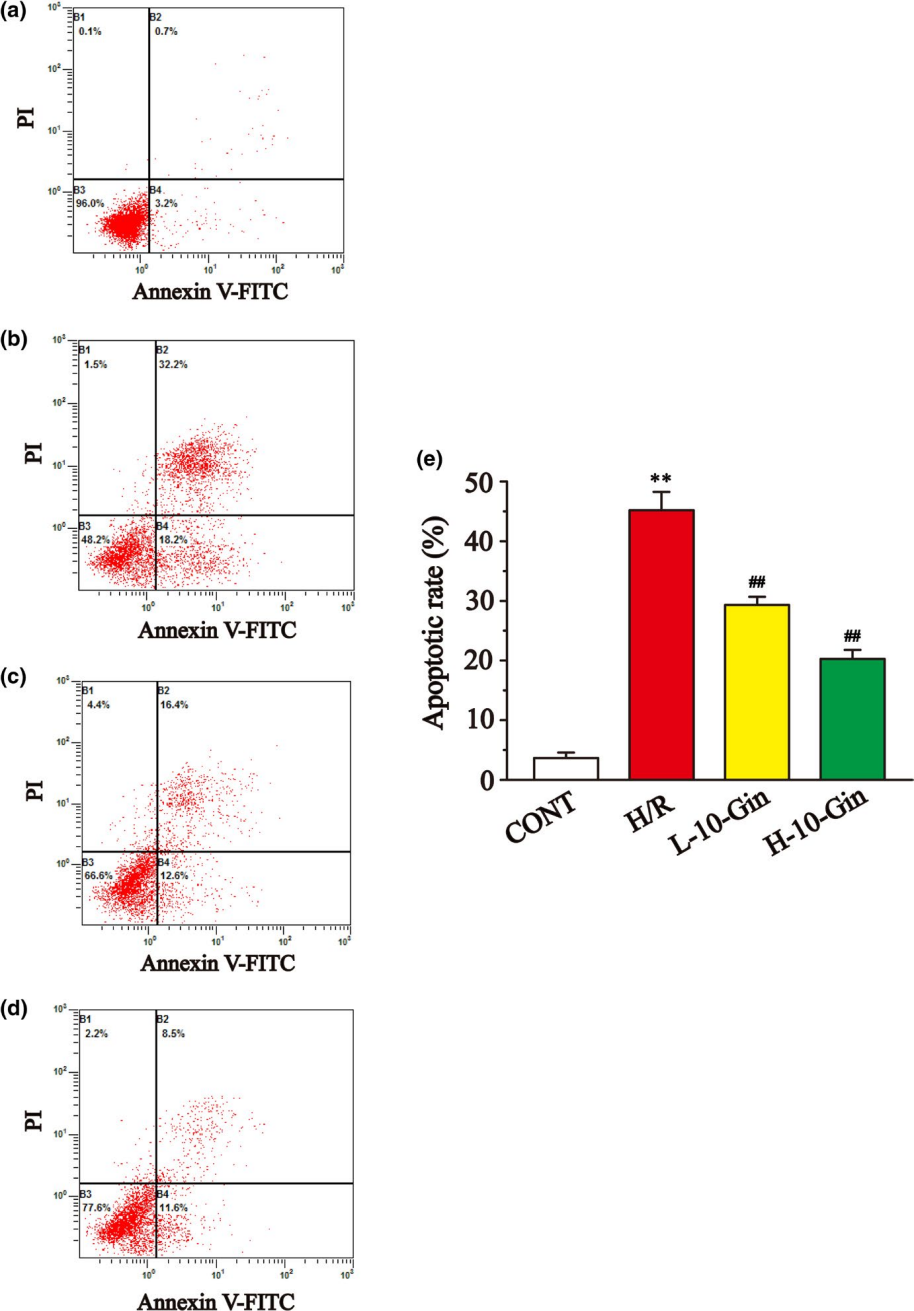
Effects of 10‐Gin on cell apoptosis of H/R cardiomyocytes by flow cytometry. Apoptotic images of different groups (a) CONT group; (b) H/R group; (c) L‐10‐Gin treatment group; and (d) H‐10‐Gin treatment group) and the ratio graph E about apoptotic rate are presented. Data are represented as mean ± *SEM*. ** *p* <.01 versus. CONT; ^##^ *p* <.01 versus. H/R group, *n* = 3

**FIGURE 9:**
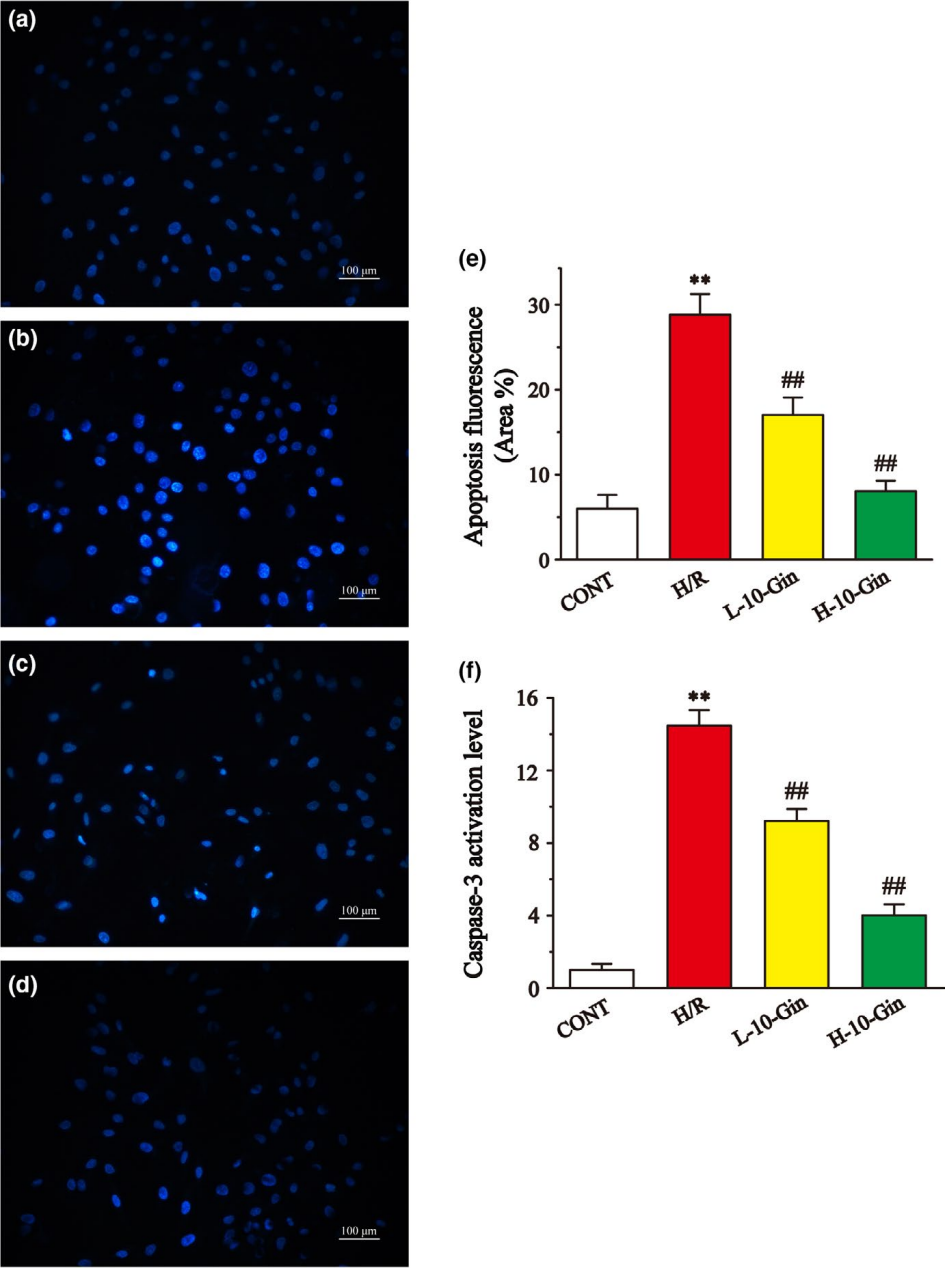
Effects of 10‐Gin on cell apoptosis of H/R cardiomyocytes by Hochest 33,258 staining and caspase‐3 kit. Apoptotic fluorescence images of different groups (a) CONT group; (b) H/R group; (c) L‐10‐Gin treatment group; and (d) H‐10‐Gin treatment group), the ratio graph (e) about apoptotic fluorescence intensity, and the graph F about caspase‐3 activity are presented. Scale bar =100 µm (200 ×). Data are represented as mean ± *SEM*. ** *p* <.01 versus. CONT; ^##^ *p* <.01 versus. H/R group, *n* = 6

Figure 8 shows the effects of 10‐Gin on apoptosis by detecting flow cytometry. The flow cytometry result shows that the apoptosis rate of the H/R group was significantly upregulated compared with the CONT group (*p* <.01), while the apoptosis rate of the 10‐Gin treatment group was remarkably lower than that of the H/R group (*p* <.01). Meanwhile, the antiapoptotic effect of 10‐Gin was further demonstrated by measuring the Hoechst‐33258 staining (Figure 9) and using a caspase‐3 kit (Figure 9f). The fluorescence intensity and caspase‐3 activity of the H/R group were significantly upregulated relative to the CONT group (*p* <.01). However, compared with the H/R group, the 10‐Gin treatment significantly decreased the fluorescence intensity and caspase‐3 activity (*p* <.01). Therefore, 10‐Gin can protect against cardiomyocyte apoptosis induced by H/R.

### 3.9 Effects of 10‐Gin on the gene and protein expressions of Wnt5a and Frizzled‐2

**FIGURE 10:**
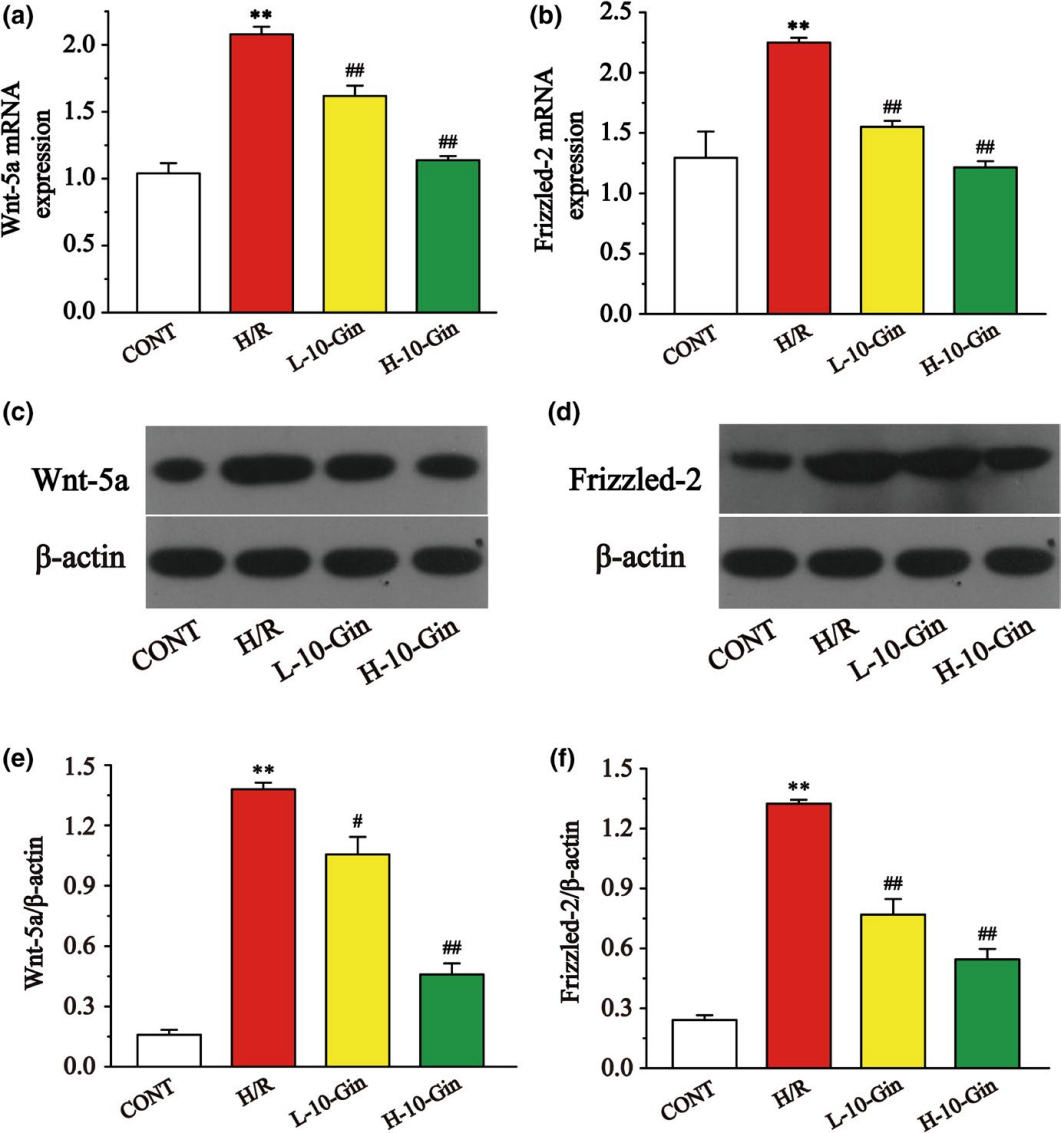
Effects of 10‐Gin on the Wnt5a (a, c, e) and Frizzled‐2 (b, d, f) gene and protein expressions. Data are represented as mean ± *SEM*. ** *p* <.01 versus. CONT; ^#^ *p* <.05, ^##^ *p* <.01 versus. H/R group, *n* = 3

RT‐PCR and Western blotting were used to detect the expression of the Wnt5a and Frizzled‐2 genes and proteins in H9c2 cardiomyocytes (Figure 10). The gene and protein expressions of Wnt5a and Frizzled‐2 in the H/R group were significantly increased compared with the CONT group (*p* <.01). However, 10‐Gin treatment was markedly decreased compared with the H/R group (*p* <.05 or *p* <.01). These results suggest that 10‐Gin has a protective effect on H9c2 cardiomyocytes by inhibiting the expression of Wnt5a and Frizzled‐2 genes and proteins.

## 4 DISCUSSION

As the main active ingredient in ginger, gingerol has been reported to have a myocardial protective effect (Namekata et al., 2008; Maier et al., 2000). However, there are no studies reporting that 10‐Gin can protect against MIRI. This study shows that 10‐Gin increased the viability of H9c2 cells in the H/R model (Figure 2d). Furthermore, we explored the protection of 10‐Gin treatment on H/R‐induced injury through oxidative stress, inflammation, and apoptosis, and its related signal pathways were also explored.

CoCl_2_ is commonly used for chemical hypoxia simulation in in vitro experiments (Jung et al., 2007). Co^2+^ in CoCl_2_ has a low affinity for oxygen, which can bind to oxygen by replacing Fe^2+^ in the hemoglobin protein porphyrin ring, causing cell hypoxia (Xiao et al., 2012). H9c2 cardiomyocytes are a kind of cardiac cell line isolated from the embryonic heart of rats with morphological characteristics similar to those of embryonic rat cardiomyocytes (Hescheler et al., 1991). H9c2 cardiomyocytes also have similar electrophysiological characteristics to rat cardiomyocytes (Eguchi et al., 2008). Therefore, the H/R model was established by treating H9c2 cardiomyocytes with 600 μmol/L CoCl_2_ for 24 hr and reoxygenating for 3 hr (Figure 2a‐c). CK and LDH are stable cytosolic enzymes that exist in cardiomyocytes (Muders et al., 2001). When the myocardial cell membrane is damaged, CK and LDH are rapidly released from the cells into the cell culture medium (Muders et al., 2001). Therefore, CK and LDH kits can be used to detect the cell damage caused by H/R. In the present study, 10‐Gin treatment significantly decreased the CK and LDH activities, proving that 10‐Gin offers protection against H/R‐induced cardiomyocyte injury (Figure 2e and 2f).

The function of the OFR scavenging system is reduced during reperfusion, while the activity of the OFR generating system is gradually enhanced, leading to a large increase in OFR, which causes serious oxidative stress damage to cardiomyocytes (Hess & Manson, 1984). SOD and CAT, as the main enzymes in the antioxidant system, can inhibit the further development of oxidative stress (Wang et al., 2017). GSH is an abundant and ubiquitous low‐molecular‐weight thiol that has been proposed to have roles in many cellular processes, including protection against the deleterious effects of ROS (Grant et al., 1998). The level of MDA could reflect the degree of lipid peroxidation, thereby indirectly reflecting the degree of cell injury (Del Rio et al., 2005). SOD could convert superoxide anion produced by lipid peroxidation into H_2_O_2_, and CAT and GSH transform H_2_O_2_ into oxygen and water, so the activities of these three enzymes are tested to explore the damage degree of the antioxidant system (Aruoma & Halliwell, 1987; Hoekstra et al., 2003). In this study, the SOD, CAT, GSH, and MDA data prove that the 10‐Gin treatment could significantly reduce the oxidative stress level, showing that 10‐Gin protects against H9c2 cardiomyocyte injury induced by H/R through antioxidant stress effects (Figure 3).

The generation of oxidative stress injury is closely related to myocardial ischemia/hypoxia (Giordano, 2005). ROS are a series of cell molecules produced in the process of oxygen metabolism and important mediators in oxidative stress injury (Del Rio et al., 2005). As the main site of ROS production, mitochondria play an important role in MIRI (Madungwe et al., 2016). During MIRI, mitochondrial function might be affected by [Ca^2+^]_i_ overload (Hurst et al., 2017). Also, the opening of mPTP is a key determinant of mitochondrial dysfunction (Weiss et al., 2003). When mPTP is turned on, free solutes and proteins can be distributed in the mitochondrial inner membrane, causing MMP to collapse (Kroemer, 1999). The opening of mPTP causes mitochondrial dysfunction, leading to ATP depletion and ultimately to cardiomyocyte apoptosis (Li et al., 2017; Whelan et al., 2010). Therefore, H/R‐induced mitochondrial dysfunction can be improved by inhibiting [Ca^2+^]_i_ overload, preventing the excessive production of ROS, blocking the opening of mPTP, increasing MMP, and reducing ATP consumption.

This study shows that the ROS of H9c2 cardiomyocytes stimulated by H/R was significantly increased compared with the CONT group (Figure 4). Meanwhile, the uptake ability of Rh123 in H9c2 cardiomyocytes of the H/R group was significantly reduced, suggesting that intracellular MMP was reduced (Figure 6). However, 10‐Gin treatment can significantly reduce the level of ROS and increase MMP. Moreover, we used Fluo‐3 a.m. fluorescent dye to measure the Ca^2+^ concentration in H9c2 cardiomyocytes and showed the [Ca^2+^]_i_ concentration through the fluorescence intensity. Our results show that 10‐Gin treatment can significantly reduce the [Ca^2+^]_i_ concentration, indicating that 10‐Gin has a protective effect on H/R‐induced [Ca^2+^]_i_ overload injury (Figure 5).

Inflammation can cause substantial cell damage and organ dysfunction, which is another important cause of MIRI (Li et al., 2002). In MIRI‐induced inflammation, TNF‐α can mediate the release of other inflammatory factors, such as IL‐6, leading to a cascade of inflammation and aggravating cell damage (Hu et al., 2016). Among these, the high concentration of IL‐6 will activate neutrophils, lymphocytes, and macrophages and induce oxidative damage to the myocardium (Jian et al., 2016). IL‐1β can induce cell apoptosis and promote cardiomyocyte remodeling (Zhu et al., 2019). In this experiment, the levels of TNF‐α, IL‐1β, and IL‐6 were measured by the ELISA method. Our results show that the 10‐Gin pretreatment effectively reduced the production of TNF‐α, IL‐1β, and IL‐6, suggesting that 10‐Gin could reduce the inflammatory response to produce myocardial protection (Figure 7).

The cascade activation of caspase is an important mechanism in cell apoptosis (Moorjani et al., 2009). Caspase‐3, as the final common pathway of apoptosis, is an important sign of apoptosis (Arumugam et al., 2006). Also, [Ca^2+^]_i_ overload can indirectly induce apoptosis by damaging mitochondria and promoting the generation of free radicals (Scarabelli et al., 2002). In this study, our flow cytometry and apoptotic fluorescence results show that the 10‐Gin treatment could significantly reduce the apoptosis of H9c2 cardiomyocytes caused by H/R (Figures 8 and 9). Also, the 10‐Gin treatment also clearly reduced the level of caspase‐3, further supporting the antiapoptotic effect of 10‐Gin (Figure 9f).

Wnt is a glycoprotein family member and an important extracellular signaling factor. As a ligand, Wnt can combine with the corresponding receptor in the Frizzled family, bringing about corresponding effects on cell differentiation and cell proliferation (De, 2011). As the cell surface receptor of Wnt5a, Frizzled‐2 combines with Wnt5a to activate the downstream transduction signal and promote the release of [Ca^2+^]_i_ (Gasser, 1988). Therefore, the Wnt5a/Frizzled‐2 pathway is also known as the Wnt/Ca^2+^ pathway. The Wnt5a/Frizzled‐2 pathway can be activated upon ischemia/anoxia stimulation (Blankesteijn et al., 1997). It is worth noting that a study has reported that when the rat Frizzled‐2 gene is ectopically expressed in the zebrafish embryo cell, the coexpression of Wnt5a is increased (Slusarski and Yang‐Snyder et al., 1997) and that the increased free Ca^2+^ comes from the Ca^2+^ pool of the mitochondria and endoplasmic reticulum. In this study, the gene and protein expression levels of Wnt5a and Frizzled‐2 were examined to demonstrate that 10‐Gin inhibits [Ca^2+^]_i_ overload through inhibiting the Wnt5a/Frizzled‐2 pathway. Our results demonstrate that 10‐Gin treatment significantly decreases the gene and protein expression levels of Wnt5a and Frizzled‐2 (Figure 10).

In this study, the H/R‐induced H9c2 cardiomyocyte injury model was used to mimic MIRI, and the cardiomyocytes were pretreated with different concentrations of 10‐Gin. The results showed that 10‐Gin attenuated oxidative stress, inflammatory response, apoptosis damage, and decreased [Ca^2+^]_i_ concentration. Although the H9c2 cell line is commonly used in in vitro studies of heart‐related diseases due to its similar current physiology, biochemistry, and other properties as primary cardiomyocytes (Zhu et al., 2017), it is still different from primary cardiomyocytes, such as cell morphology, cell contractility, and gene expression (Hescheler et al., 1991; Xie et al., 2016; Zhu et al., 2017). In addition, the heart is regulated by many mechanisms including neurotransmitters and body fluids in in vivo, the mechanisms are complex and cannot be manifested in in vitro. The investigations of the present study are limited to the cellular level, so in vivo experiments should be conducted to further explore the protective effects of 10‐Gin on MIRI.

## 5 CONCLUSIONS

In conclusion, 10‐Gin can attenuate the injury caused by H/R and reduce oxidative stress, [Ca^2+^]_i_ overload, inflammation, and apoptosis, which is related to the inhibition of the Wnt5a/Frizzled‐2 pathway. Our research provides an experimental basis and new treatment strategies for MIRI. However, further research will be needed before these treatments can be used in clinics.

## CONFLICTS OF INTEREST

The authors declare that they do not have any conflict of interest.

## ETHICAL APPROVAL

This study does not involve any human or animal testing.

## Data Availability

The datasets used and/or analyzed during the current study are available from the corresponding author on reasonable request.
